# Quantitative differences, qualitative outcomes

**DOI:** 10.7554/eLife.04869

**Published:** 2014-10-29

**Authors:** Giulia Pollarolo, Cayetano Gonzalez

**Affiliations:** 1 **Giulia Pollarolo** is a Juan de la Cierva Fellow and is in the Institute for Research in Biomedicine, Barcelona, Spain; 2 **Cayetano Gonzalez** is in the Institució Catalana de Recerca i Estudis Avançats (ICREA) and the Institute for Research in Biomedicine, Barcelona, Spaingonzalez@irbbarcelona.org

**Keywords:** quiescence, prospero, neural progenitor, *D. melanogaster*

## Abstract

Fruit fly neuroblasts can either self-renew, rest or take on a specialized form, depending on the levels of a protein called Prospero.

**Related research article** Lai SL, Doe CQ. Transient nuclear prospero induces neural progenitor quiescence. *eLife* **3**:e03363. doi: 10.7554/eLife.03363

**Image** Mutant fly embryo neuroblasts that cannot produce the protein Prospero do not enter a resting state known as quiescence (as shown by the blue, red and green cells above the dashed line)

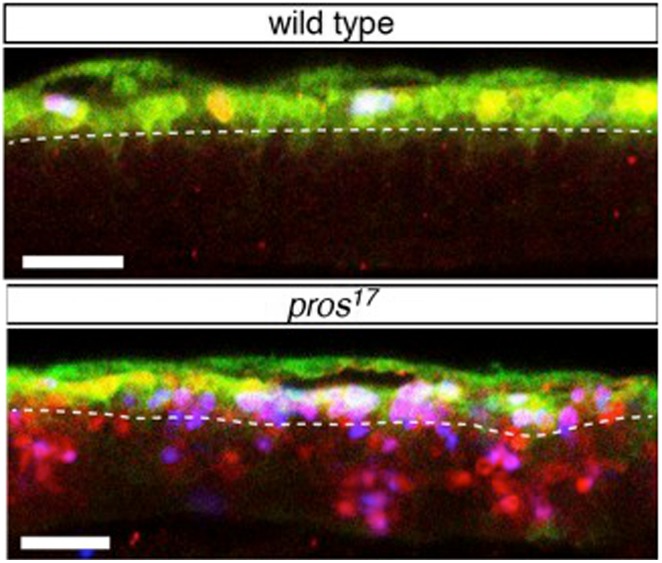

Quiescence (from the Latin meaning ‘to become quiet’) is a state during which cells stop progressing through the cell cycle and cease to divide. However, quiescent cells retain the ability to start moving through the cell cycle again. Programmed quiescence of many types of cells, including stem cells, is essential for organisms to develop normally. Quiescence is also crucial for maintaining tissues in a healthy condition in adults. Unveiling the molecular machinery that regulates quiescence is, therefore, of paramount importance for understanding development and disease.

Insight into how quiescence is regulated has been obtained through studies on neuroblasts—a type of brain stem cell—in the fruit fly *Drosophila*. The neuroblasts become quiescent during the late stages of embryonic development and resume growing and dividing as the larva hatches. Studies carried out over the last few years have revealed that these cells exit the quiescent state in response to a nutritional stimulus (Chell and Brand, 2010; Sousa-Nunes et al., 2011).

Now, in *eLife*, Sen-Lin Lai and Chris Doe at the University of Oregon report that the amount of a protein called Prospero controls when neuroblasts enter quiescence (Lai and Doe, 2014). Unlike cells that divide symmetrically to produce two identical cells, neuroblasts divide asymmetrically to make a new neuroblast and a ganglion mother cell. The mature cells that make up the central nervous system develop from these ganglion mother cells. Prospero is one of several molecules that, by being unequally divided between the new neuroblast and the ganglion mother cell, give each cell its identity.

In the developing *Drosophila* central nervous system, Prospero is well known for its essential roles in repressing neuroblast self-renewal, stopping neuroblasts proliferating, and promoting the differentiation of the neuroblast into a final specialized cell type (Choksi et al., 2006). There is also evidence showing that Prospero can temporarily pause the cell cycle of a certain type of cell that generates the glial cells that support the neurons in the brain (Griffiths and Hidalgo, 2004).

The new results obtained by Lai and Doe demonstrate that, at the end of their proliferative phase, embryonic neuroblasts produce a brief, low-level pulse of Prospero that causes them to become quiescent. In addition, Lai and Doe found that introducing similar pulses of Prospero into cells experimentally can drive proliferating embryonic neuroblasts into premature quiescence (Figure 1).

**Figure 1.**
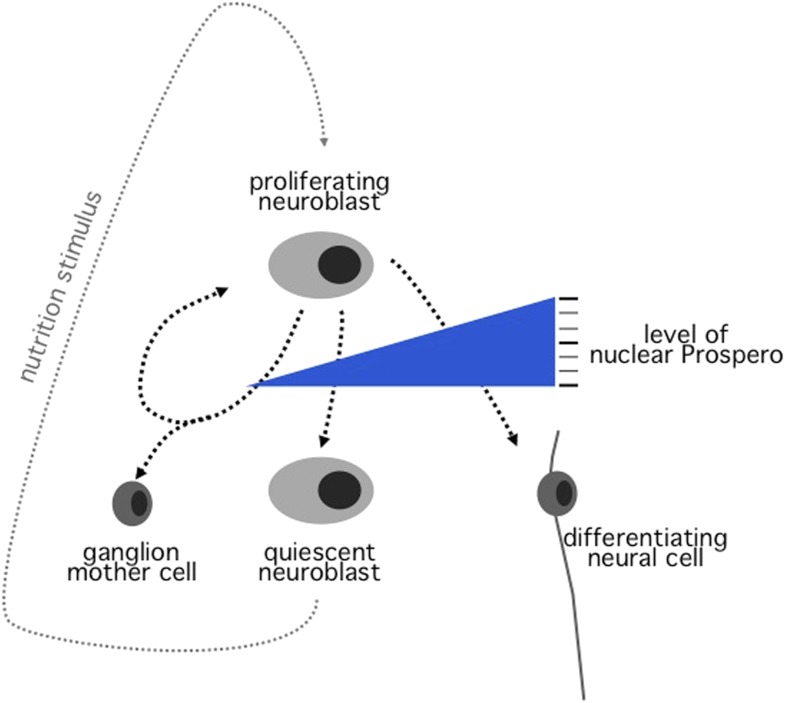
In neuroblasts, the amount of a protein called Prospero controls the behaviour of the cell. Drosophila embryonic neuroblasts undergo asymmetric cell division—which produces a ganglion mother cell and a renewed neuroblast—when the level of Prospero (indicated by the blue triangle) is minimal (left). Low levels of Prospero drive proliferating neuroblasts into quiescence (middle). Quiescent neuroblasts can become proliferative again in response to a nutrition-dependent signal produced by an organ in the larva called the fat body. High levels of Prospero force proliferating neuroblasts to differentiate and become a specialized neural cell (right).

The conclusion that Prospero is necessary for neuroblast quiescence is supported by evidence that appeared to show that a large number of neuroblasts fail to quiesce in mutant *Drosophila* embryos that are unable to produce Prospero. However, a lack of Prospero also results in the formation of neuroblast-like ganglion mother cells, which do not quiesce (Choksi et al., 2006; Lee et al., 2006). Therefore, Lai and Doe needed to implement a method that could tell apart neuroblast-like cells from bona fide neuroblasts.

To this end, taking advantage of the wealth of knowledge and tools developed over the years in *Drosophila* research, Lai and Doe made use of reporter genes that indicate when Notch signalling occurs. Notch is a protein that helps neighbouring cells to signal to each other. Unlike neuroblasts, cells derived from ganglion mother cells express a protein that inhibits Notch signalling. Therefore, if a neuroblast-like cell displays Notch signalling activity, it must be a neuroblast. This meant Lai and Doe were able to confirm that the cells they investigated were neuroblasts, and that a lack of Prospero does prevent quiescence.

The conclusion that Prospero is sufficient for quiescence is supported by experiments that introduced a low-level pulse of Prospero to cells, which resulted in many neuroblasts becoming quiescent. Here again, the multitasking nature of Prospero made experiments technically difficult, as high levels of Prospero can cause neuroblasts to differentiate and become a specialized cell (Choksi et al., 2006).

To rule out this possibility, Lai and Doe delivered a short pulse of Prospero using the TARGET method (McGuire et al., 2003), which controls when and where genes are expressed. Importantly, once Prospero levels returned to normal background levels, the resulting quiescent neuroblasts started proliferating again, confirming that neuroblasts exposed to a short pulse of Prospero do indeed become quiescent.

Lai and Doe have therefore established that different levels of Prospero control different behaviours in neuroblasts. When the level of Prospero is minimal, neuroblasts can divide asymmetrically to form a ganglion mother cell; a low-level pulse of Prospero drives proliferating neuroblasts into quiescence; and high levels force the neuroblasts to differentiate into a specialized cell type (Figure 1).

In mice, Prox1—the vertebrate form of Prospero—has several roles during retinal development: for example, it enables the embryonic cells that produce the different cells in the retina to exit the cell cycle; it is required for the specification of the cells that are responsible for allowing eyes to adjust to changing light conditions; and it is essential for maturing the cells that transmit signals from the photoreceptors. The analysis of individuals who had one non-functional copy of the *Prox1* gene suggests that some of these functions may depend on how much Prox1 is present (Dyer, 2003). Prox1 has been linked to a variety of cancer types in humans in the last few years (Elsir et al., 2012).

Although so far limited to *Drosophila* embryonic neuroblasts, the results reported by Lai and Doe add a new case to the list of qualitatively different outcomes driven by quantitative differences. This is abundantly exploited in different contexts during development and, perhaps, might also be relevant for disease.
